# Rate-Dependent and Relaxation Properties of Porcine Aortic Heart Valve Biomaterials

**DOI:** 10.1109/OJEMB.2020.3002450

**Published:** 2020-06-15

**Authors:** CHRISTOPHER NOBLE, MICHAEL KAMYKOWSKI, AMIR LERMAN, MELISSA YOUNG

**Affiliations:** Department of Cardiovascular Medicine, Mayo Clinic, Rochester, MN 55905 USA

**Keywords:** Aortic valve replacement, Biaxial tension, rate-dependency, relaxation testing, tissue engineered heart valve

## Abstract

**Objective:**

This work evaluates the rate-dependent and relaxation properties of native porcine heart valves, glutaraldehyde fixed porcine pericardium, and decellularized sterilized porcine pericardium.

**Methods:**

Biaxial tension testing was performed at strain-rates of 0.001 s^−1^, 0.01 s^−1^, 0.1 s^−1^, and 1 s^−1^. Finally, relaxation testing for 300 s was performed on all heart valve biomaterials.

**Results:**

No notable rate-dependent response was observed for any of the three biomaterials with few significant differences between any strain-rates. For relaxation testing, native tissues showed the most pronounced drop in stress and glutaraldehyde the lowest drop in stress although no tissues showed anisotropy in the relaxation.

**Conclusions:**

Increasing the strain-rate of the three biomaterials considered does not increase the stress within the tissue. This indicates that there will not be increased fatigue from accelerated wear testing compared to loading at physiological strain-rates as the increase strain-rates would likely not significantly alter the tissue stress.

## I. INTRODUCTION

Currently, FDA and ISO guidance standards exist for heart valve performance for use in humans that require detailed in vivo and in vitro tests [1]. Accelerated wear testing (AWT) evaluates the valve performance at frequencies that are far higher than physiological allowing many years of equivalent real-time fatigue to be performed in months. However, increased frequency results in increased tissue strain rates which may lead to increased stress in the tissues compared to tests conducted at physiological frequencies due to the time dependent mechanical behavior of the tissue. Such increased stress concentrations may lead to increased tissue fatigue and risk of failure which may not be seen if tests were performed at physiological frequencies. In this work we aimed to investigate the time dependent behavior of three heart valve biomaterials; native porcine aortic valve, glutaraldehyde fixed porcine pericardial tissue (similar to that used in bioprosthetic heart valves), and our in house decellularized-sterilized porcine pericardium utilized for TEHVs. Viscoelastic behavior of native aortic heart valves has been previously evaluated by Stella *et al.* using strain rate, creep, and stress relaxation experiments on porcine tissues on a biaxial tensile test machine [2]. They performed equibiaxial tension to a membrane tension of 60 N/m and loaded and unloaded samples in half cycles time of 1, 0.5, 0.1, and 0.05 s. Creep and relaxation tests were performed with a rise time of 100 ms. Anssari-Benam *et al.* have also performed variable strain rate tests on native porcine aortic heart valves using biaxial tension under various loading protocols [3], [4]. They performed biaxial tension testing with equibiaxial displacement and a 3:1 ratio in favor of the radial direction at strain rates of 0.001 s^−1^, 0.01 s^−1^, 0.1 s^−1^, and 1 s^−1^. In addition, they performed tests with and without preconditioning to evaluate the effect of this on valve viscoelasticity. For glutaraldehyde fixed tissues Duncan and Boughner conducted uniaxial tensile tests and stress-relaxation tests on glutaraldehyde treated bovine pericardium at two different extension rates with testing performed at 3 and 30 mm/s [5]. Additionally, the viscoelastic properties of fixed bovine pericardium have been evaluated using creep and stress relaxation testing [6]–[8]. However, investigations of viscoelastic properties for TEHV are rare with studies devoting focus on the considerable problem of the elastic properties and appropriate constitutive models to accurately reflect the complex mechanical environment including fatigue and remodeling. Mendoza-Novelo *et al.* have previously investigated the viscoelastic properties of decellularized bovine pericardium using uniaxial stress-relaxation testing with tests performed to 100 kPa and a recovery time of 100 s [9]. However, no one has characterized the viscoelastic properties of decellularized porcine pericardium using biaxial stress-relaxation testing.

## II. RESULTS

### A. HEART VALVE BIOMATERIAL RATE-DEPENDENCY

**FIGURE 1.**
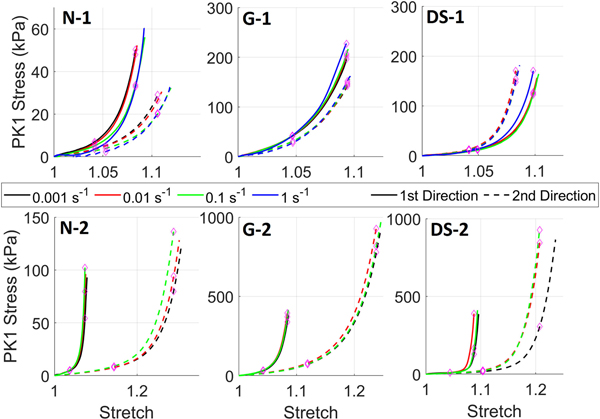
First Piola-Kirchhoff (PK1) stress versus stretch curves for mean native aortic valve cusp data for the first loading protocol (10% applied equibiaxial strain; N-1) and the second loading protocol (30% applied strain in x_1_ and 10% applied strain in x_2_; N-2). Mean glutaraldehyde fixed (G-1 and G-2) and decellularized-sterilized pericardium (DS-1 and DS-2) curves are also shown. Purple diamonds illustrate points where stress was found at λ_c_ and λ_c_/2.

Fig. 1 shows the mean stress versus stretch for the three tissue groups for the two loading regimes at the four strain-rates. Overall, no clear rate-dependent response can be seen for either testing regime. However, for the first testing regime the fastest strain rate does show a steeper path compared to the other test speeds for the native and glutaraldehyde fixed tissues. The salient features for each tissue are described below:

#### Native Tissues:

**FIGURE 2.**
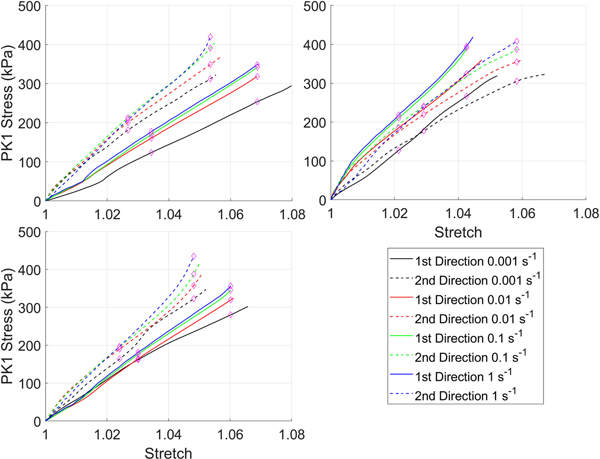
First Piola-Kirchhoff (PK1) stress versus stretch curves for silicone rubber tested without the preconditioning protocol.

No incremental rate dependency can be seen; both 0.01 and 1 s^−1^ curves show a steeper response for both testing directions but the 0.1 s^−1^ curve shows a less steep response for both testing directions, although the final stress is higher. This is also reflected in the statistical analysis where it can be seen that at λ_c_ there was no significant differences in stress for either direction. However, at λ_c_/2 for x_1_ the 1 s^−1^ stress value was statistically significant when compared to the other values (Table 1). For the second testing regime (3:1 displacement) there is increasing curve steepness with increasing strain-rate for both testing directions indicating some rate-dependency (Fig. 1 N-2) although this was not statistically significant in either direction at λ_c_ or λ_c_/2 (Table 1).

#### Glutaraldehyde Fixed Pericardium:

For the first testing regime the curves show similar profiles for all strain rates although the stress is approximately 25% greater for the 1st direction (Fig. 1 G-1). Additionally, there is little difference between 0.001 and 0.01 s^−1^ strain-rates and only a small difference between the 0.1 and 0.001 s^−1^ strain-rates with the only significant difference for λ_c_ (Table 1) and the x_1_ direction. However, the 1 s^−1^ strain-rate does show a stiffer response for both testing directions. In the second testing regime, there was no prominent difference between the strain-rates tested (Fig. 1 G-2) and there was no statistical significance in either direction for at either λ_c_ or λ_c_/2 (Table 1).

#### Decellularized Sterilized Pericardium:

There was very little difference between the curve profiles for either testing direction barring the fastest strain rate, but curves from the 1st direction extend further resulting in a higher maximum stress (Fig. 1 DS-1). Furthermore, barring the fastest strain-rate (1 s^−1^) for the 1st testing direction, the data from the four strain-rates is difficult to distinguish. This is highlighted by the statistical comparisons where only the difference between 0.01 s^−1^ and 0.1 s−^1^ strain-rates was significant for λ_c_/2 and x_1_ direction only (Table 1). Finally, for the second testing regime the curves are closely clustered barring the curve for the 0.001 s^−1^ strain-rate which is markedly separate from the other curves generated from the 2^nd^ testing direction (Fig. 1 DS-2). However, there were no significant differences between any of the strain-rates in either direction at either λ_c_ or λ_c_/2 (Table 1).

### B. SILICONE RUBBER RATE-DEPENDENCY

**FIGURE 3.**
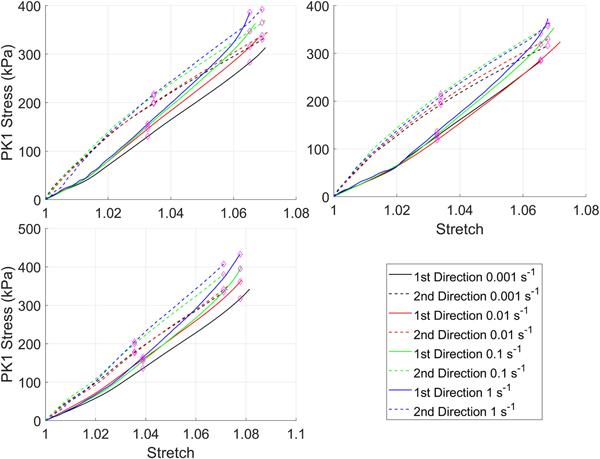
First Piola-Kirchhoff (PK1) stress versus stretch curves for silicone rubber tested with the preconditioning protocol.

Stress-strain curves for the silicone rubber with and without the preconditioning protocol are in Figs. 2 and 3 respectively. For clarity, all test data from all samples have been shown. Overall, regardless of whether the preconditioning protocol was performed there was a consistent rate-dependent response. However, the magnitude of the difference is small, and for some samples, the curves from different strain-rates overlap as a result of this. This is reflected in the statistical comparison (Table 2) where almost all curves had differences that were statistically significant at λ_c_ regardless of whether preconditioning was performed.

### C. HEART VALVE BIOMATERIAL RELAXATION TESTING

**FIGURE 4.**
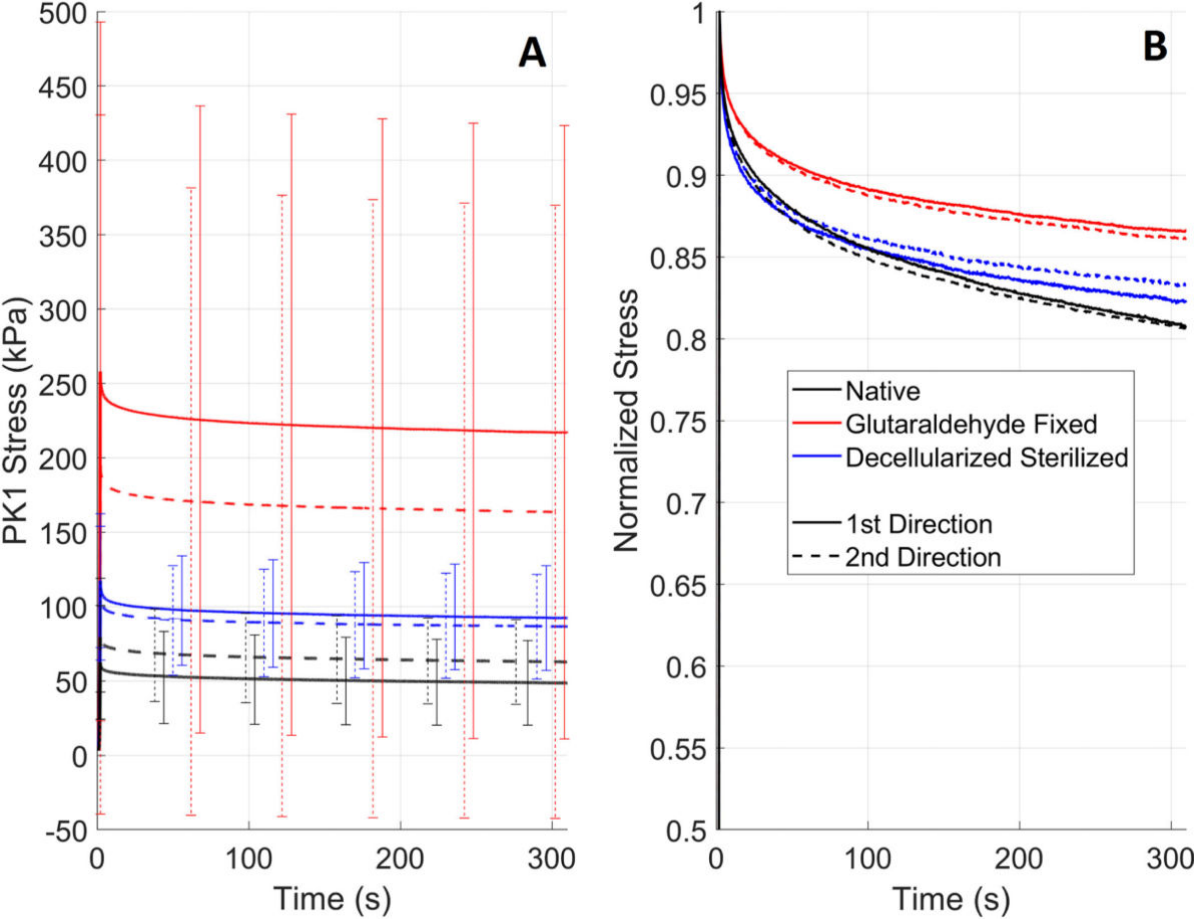
Mean relaxation curves for native valvular cusps, glutaraldehyde fixed pericardium, and decellularized-sterilized pericardium with first Piola-Kirchhoff (PK1) Stress (A) or stress normalized (B).

Relaxation of the three tested tissue types can be seen in Fig. 4A and curves normalized by the maximum stress can be seen in Fig. 4B to allow comparison of the relaxation between the tissues irrespective of the tissue strength. A summary for each tissue type is given below:

#### Native Tissues:

Compared to other tissues the peak stress is lowest for both directions. Like for the biaxial tension test in Fig. 1 (N-1), the 1st direction shows the highest peak stress. Both curves show significant relaxation, dropping to almost 80% of the peak value in 300 s.

#### Glutaraldehyde Fixed Pericardium:

The difference between peak stresses is the largest of the three tissues despite the biaxial tension test showing lower anisotropy than native tissues (Fig. 4A). The peak stress is also highest for both directions. The relaxation is lowest with a drop to approximately 87% of the peak value.

#### Decellularized Sterilized Pericardium:

The peak stresses of both directions lie in the middle of the three tissues, and the difference between the peak stresses is the smallest which aligns well with the biaxial tension data in Fig. 1 (DS-1). The normalized relaxation curves also lie in the middle of the three tissues with a reduction in stress from the peak value of approximately 83%.

## III. DISCUSSION

AWT evaluates aortic valve replacement fatigue with physiological loading at 20 times the in vivo frequency. If rate-dependent behavior is present within a heart valve biomaterial then it may be expected that during AWT, the valve would experience higher stresses and may fatigue faster than in vivo. However, for the two replacement biomaterials tested here (glutaraldehyde treated and decellularized-sterilized porcine pericardium) there was negligible rate-dependency observed indicating that neither of these materials fatigue differently during AWT compared to testing at physiological frequency. Physiological in vivo strain rates for aortic valvular tissues have been estimated to be approximately 4.4 s^−1^ and 12.4 s^−1^ in the circumferential and radial directions respectively [10]. For this to be replicated in vitro extensions rates up to 50 to 150 mm/s would be required [11], [12]. Furthermore, as AWT frequency is approximately 20 times higher than physiological conditions the strain-rates would be expected to be proportionally higher. Performing biaxial testing for small displacements at these speeds proposes numerous technical challenges and thus lower testing speeds were chosen. However, there may be a rate-dependent response at these higher strain-rates and so care must be taken when interpreting the results of this study.

In addition, relaxation testing was performed to further evaluate the tissue viscoelastic behavior and to further evaluate performance during AWT. Glutaraldehyde fixed pericardium demonstrated the least relaxation and thus may take longer to relax to its resting state. This has implications for AWT as the valve may have a higher preload for each subsequent cycle and thus fatigue faster [8]. Compared to previous studies, low rate-dependency of glutaraldehyde fixed pericardium has been observed previously with tissue tested at 30 mm/s and 3 mm/s showing similar elastic modulus [5]. For decellularized pericardium there was little difference in relaxation compared to the native tissues in this study which is supported by Mendoza-Novelo *et al.* where decellularized bovine pericardium has been shown to have similar relaxation response to native tissues [9].

Native tissues also demonstrated limited rate-dependency with only the fastest strain-rate showing a significant difference compared to the three other strain-rates and only in the first testing direction. Additionally, the relaxation testing demonstrated significant reductions in stress after 300 s. Comparable findings for viscoelastic behavior of porcine aortic valve leaflets have been identified by Stella *et al.* where they similarly observed relaxation and limited rate-dependency but also limited creep [2]. They postulated that these differences may be a result of interactions between small proteoglycans and collagen fibrils leading to viscoelasticity under non-physiological loading but an intrinsic elastic response of the collagen fibrils. In contrast to the results of this study and those of Stella *et al.*, Annsari Behnam *et al.* found significant rate-dependent behavior of porcine aortic cusps [3]. Samples were tested at 0.001 s^−1^ and 1 s^−1^ with and without preconditioning and with differing loading regimes. Compared to the native tissues tested in this study all samples showed notable rate dependency with statistical analysis showing significant increases in the stress-stretch curve gradient at all points probed for both testing directions regardless of whether preconditioning was performed. Both this study and the work by Stella *et al.* utilized preconditioning which has been argued to remove rate-dependency [13], [14]. However, in the study by Annsari-Behnam *et al.* preconditioning appeared to increase the stiffness of the tissue tested at the slowest strain-rate and decrease the stiffness of the tissue at the fastest strain-rate although as mentioned the differences between the two strain-rates remained statistically significant. Similar test protocols were utilized in this study and testing of silicone rubber showed statistically significant differences between almost all strain rates. Additionally, there were significant differences between all strain-rates and the 1 s^−1^ strain-rate at λ_c_/2 for the first test direction which may indicate some rate-dependency. A possible explanation for the differences may be the preconditioning protocol, in this study 10 loading-unloading cycles of the final loading protocol (i.e. 10% applied strain in each direction for the equibiaxial protocol and 30% applied strain in x_1_ and 10% applied strain in x_2_) were applied. In contrast, in the manuscript by Ansarri-Benam *et al.* 25 loading-unloading cycles were performed to 0.5 N. Therefore, the force controlled preconditioning protocol may explain the difference in rate-dependency. The magnitude of stresses observed by Anssari-Benam *et al.* for native tissues were considerably higher than in this study but similar magnitudes to those found here have been recorded elsewhere for both native tissues and glutaraldehyde fixed porcine pericardium [15]–[19].

## IV. CONCLUSION

The current means for evaluating replacement heart valve longevity is AWT. However, the higher strain rates on the leaflets may increase the mechanical stress leading to increased wear compared to *in vivo* loading. This work evaluated the viscoelasticity of three heart valve materials: native porcine heart valves, glutaraldehyde-fixed porcine pericardium, and our pericardial TEHV material. Biaxial tension testing was performed for equibiaxial displacement and with unequal displacement at a ratio of 3:1 non-fibrous to fibrous direction at strain-rates of 0.001 s^−1^, 0.01 s^−1^, 0.1 s^−1^, and 1 s^−1^. In addition, to verify the testing procedure the same test protocol was performed for silicone rubber. Finally, relaxation testing for 300 s was performed on all biomaterials. No notable rate-dependent response was observed for any of the three biomaterials with few significant differences in stress between any strain-rates. By contrast, the silicone rubber showed statistically significant rate-dependency between almost all strain rates, verifying the test protocol does indeed probe rate-dependency. For relaxation testing, when normalizing for peak stress, native tissues showed the most pronounced drop in stress and glutaraldehyde the lowest drop in stress. Therefore, these results indicate that the increased strain-rates observed during accelerated wear testing would likely not significantly alter the tissue stress and the resulting fatigue data compared to testing at physiological strain-rates. However, there may be a rate-dependent response at higher strain-rates equivalent to those found physiologically or in AWT.

## V. MATERIALS AND METHODS

The tissue groups tested were native porcine valves, decellularized-sterilized porcine pericardium, and glutaraldehyde fixed porcine pericardium. Porcine valves were chosen due to their relative availability compared to human tissues. Details on the tissue preparation can be found in the supplementary materials.

**FIGURE 5.**
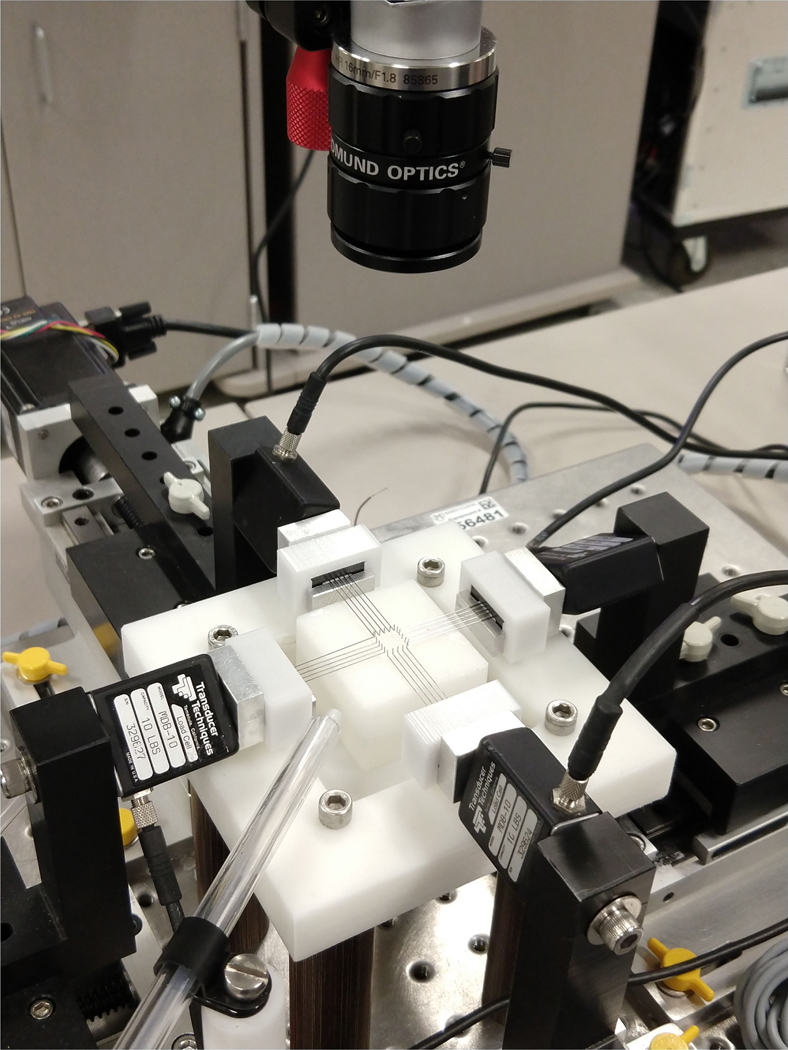
Tissue test apparatus showing the four independent arms, the biorakes, and the camera to record marker positions.

An in-house biaxial setup, with four linear actuators controlled independently by position was used for mechanical testing [20]. Force measurement was conducted by four 44.5 N load cells attached to each moving stage, two in the x_1_ direction and two in the x_2_ direction which are connected to the arms holding the testing rakes (Fig. 5; Cellscale, Ontario, Canada). Porcine cusps were cleaned and were mounted on the biaxial machine such that the circumferential direction was aligned in the x_1_ direction of the machine. Prior to testing, pericardium samples were cut into square pieces ensuring the prominent fiber direction was aligned with an edge of the square, and like for native cusps, aligned in the x_1_ direction of the machine. Five rake prongs, with tine spacing of 1.7 mm, were used to secure the tissues. The rakes were positioned to give an effective testing size of 10 mm × 10 mm. Samples were moistened using a flow system utilizing a flow-loop that ensured 37 °C saline solution was pumped around the sample and collected at the testing system base to be heated and recycled. Stretch measurement was performed with the same method as in our previous work [20] and is described in the supplementary materials.

Tissue time dependency and the testing regime was based upon work by Annsari-Benam *et al.*, Stella *et al.*, and through our initial studies [2], [3]. Testing was performed on native porcine cusps (n = 11), native porcine decellularized porcine pericardium (n = 12), and glutaraldehyde fixed pericardium (n = 12). Additional testing was also performed on silicone rubber (0.8 mm thickness polysiloxane, McMaster-Carr USA) with and without the preconditioning protocols (n = 3 for both conditions) [21], [22] in order to verify that the testing procedure correctly probed the tissue rate-dependency. First, preconditioning was performed for 10 cycles of 10% applied strain (defined as the applied displacement over the effective test length in each direction). Then, a small pre-load of 0.03 N was applied to hold the sample in tension before testing, followed by the nine small markers being placed on the sample. Equibiaxial displacements were then applied up to 0.5 mm to give an equivalent applied strain of 10% at four strain rates, 0.001 s^−1^, 0.01 s^−1^, 0.1 s^−1^, and 1 s^−1^. The following displacements were applied at a ratio of 1:3 x_1_ to x_2_ such that the larger displacement was in the direction perpendicular to the prominent fiber direction of the tissues, equivalent to 30% applied strain in x_2_ and 10% applied strain in x_1_. This was performed at three strain rates; 0.001 s^−1^, 0.01 s^−1^, and 0.1 s^−1^ in the x_1_ direction and at 0.003 s^−1^, 0.03 s^−1^, and 0.3 s^−1^ in the x_2_ direction (to ensure the maximum displacement in each direction occurred at the same time). As the testing arrangement was not accurate at the fastest strain rate in the x_2_ direction (3 s^−1^) this testing speed was not evaluated. Initial studies found that a second preconditioning protocol had to be applied of 30% applied strain in x_2_ and 10% applied strain in x_1_ for 10 cycles prior to the second loading protocol. Briefly, the initial studies found that if this preconditioning was not applied, the sample was strongest for the slowest strain rate then got weaker as strain rates increased indicating that testing order was most likely affecting the mechanical responses. The preconditioning protocols utilized here removed this effect.

Additionally, to further probe the viscoelastic behavior relaxation testing was performed. The same tissues were evaluated (n = 10 for all) and were first preconditioned with 10 cycles of 10% applied strain then the samples were pulled to 10% applied strain and held for 5 minutes with the force recorded. Samples were also kept moistened at 37 °C using the flow loop described previously.

The mechanical test data was compared by a statistical analysis described in the supplementary materials.

## Supplementary Material

Supplementary Materials

Supplementary Figure 1

Supplementary Figure 2

Supplementary Figure 3

## Figures and Tables

**TABLE 1.** Statistical Comparison of Rate-Dependency Data

		p Value					
		Native		Fixed		DS	
	Comparison	X_1_	X_2_	X_1_	X_2_	X_1_	X_2_

Equibiaxial Displacement (λ_c_/2)	0.001 **s**^−1^ – 0.01 **s**^−1^	0.993	0.958	0.999	1	0.862	0.66
	0.001 **s**^−1^ – 0.1 **s**^−1^	0.998	0.982	0.985	0.997	0.888	0.484
	0.001 **s**^−1^ – 1 **s**^−1^	**0.011**	0.328	0.327	0.837	0.144	0.975
	0.01 **s**^−1^ – 0.1 **s**^−1^	0.969	0.818	0.996	0.999	1	0.992
	0.01 **s**^−1^ – 1 **s**^−1^	**0.021**	0.619	0.394	0.866	**0.025**	0.406
	0.1 **s**^−1^ – 1 **s**^−1^	0.006	0.173	0.524	0.918	0.029	0.263
	0.001 **s**^−1^ – 0.01 **s**^−1^	0.873	0.743	0.848	0.924	0.956	0.968
Equibiaxial Displacement (λ_c_)	0.001 **s**^−1^ – 0.1 **s**^−1^	0.981	0.993	0.036	0.518	0.988	0.998
	0.001 **s**^−1^ – 1 **s**^−1^	0.137	0.937	0.099	0.907	0.998	0.726
	0.01 **s**^−1^ – 0.1 **s**^−1^	0.668	0.582	0.204	0.873	0.837	0.993
	0.01 **s**^−1^ – 1 **s**^−1^	0.472	0.973	0.412	0.571	0.988	0.936
	0.1 **s**^−1^ – 1 **s**^−1^	0.061	0.831	0.971	0.186	0.956	0.826
3x_2_: x_1_ Displacement (λ_c_/2)	0.001 s^−1^ – 0.01 s^−1^	1	0.971	0.983	0.999	0.726	0.631
	0.001 **s**^−1^ – 0.1 **s**^−1^	0.99	0.951	0.96	0.912	0.442	0.92
	0.01 **s**^−1^ – 0.1 **s**^−1^	0.988	0.855	0.995	0.928	0.887	0.856
3x_2_: x_1_ Displacement (λ_c_)	0.001 **s**^−1^ – 0.01 **s**^−1^	0.827	0.966	0.577	0.937	0.734	0.243
	0.001 **s**^−1^ – 0.1 **s**^−1^	0.379	0.891	0.127	0.997	0.485	0.962
	0.01 **s**^−1^ – 0.1 **s**^−1^	0.73	0.759	0.582	0.961	0.913	0.153

Calculated p values from mechanical test data on heart valve biomaterials from a multi-comparison test following a one-way ANOVA analysis. Native is native valvular cusps, fixed is glutaraldehyde fixed porcine pericardium, and DS is decellularized-sterilized porcine pericardium. The stress across all four strain rates was found at either λ_c_ or λ_c_/2 where λ_c_ is the lowest maximum stretch across the four strain rates.

**TABLE 2.** Silicone Rubber Rate-Dependency Statistical Analysis

		p Value					
		All		Preconditioned		Not Preconditioned	
	Comparison	X_1_	X_2_	X_1_	X_2_	X_1_	X_2_

Equibiaxial Displacement (λ_c_/2)	0.001 **s**^−1^ – 0.01 **s**^−1^	0.384	0.521	0.347	0.999	0.131	0.509
	0.001 **s**^−1^ – 0.1 **s**^−1^	0.107	0.054	0.118	0.476	**0.024**	0.229
	0.001 **s**^−1^ – 1 **s**^−1^	0.038	0.06	0.021	0.433	**0.012**	0.265
	0.01 **s**^−1^ – 0.1 **s**^−1^	0.864	0.529	0.849	0.558	0.634	0.908
	0.01 **s**^−1^ – 1 **s**^−1^	0.575	0.559	0.252	0.512	0.362	0.943
	0.1 **s**^−1^ – 1 **s**^−1^	0.953	1	0.629	1	0.949	0.999
	0.001 **s**^−1^ – 0.01 **s**^−1^	**<0.001**	**0.044**	0.087	0.357	**0.003**	**0.013**
Equibiaxial Displacement (λ_c_)	0.001 **s**^−1^ – 0.1 **s**^−1^	**<0.001**	**<0.001**	**<0.001**	**<0.001**	**<0.001**	**<0.001**
	0.001 **s**^−1^ – 1 **s**^−1^	**<0.001**	**<0.001**	**<0.001**	**<0.001**	**<0.001**	**<0.001**
	0.01 **s**^−1^ – 0.1 **s**^−1^	**<0.001**	**<0.007**	**0.025**	**0.002**	**0.02**	**0.027**
	0.01 **s**^−1^ – 1 **s**^−1^	<0.001	**<0.001**	**<0.001**	**<0.001**	**0.008**	**<0.001**
	0.1 **s**^−1^ – 1 **s**^−1^	0.073	**0.039**	**0.028**	**0.033**	0.882	**0.043**

Calculated p values from mechanical test data on silicon rubber from a multi-comparison test following a one-way ANOVA analysis. The stress across all four strain rates was found at either λ_c_ or λ_c_/2 where λ_c_ is the lowest maximum stretch across the four strain rates.

## IMPACT STATEMENT

No observed mechanical rate-dependency for both glutaraldehyde fixed and decellularized pericardium despite notable tissue relaxation. Indicates no increased stress on tissue during accelerated wear testing compared to physiological loading.
