# Supplementary material for: Rate-Dependent and Relaxation Properties of Porcine Aortic Heart Valve Biomaterials
Source: IEEE Open J Eng Med Biol. 2020 Jun 15;1:197–202. doi: 10.1109/OJEMB.2020.3002450 (PMC7971416; doi:10.1109/OJEMB.2020.3002450)
Supplement: Supplementary file 1 [file supp1-3002450.docx]

**Supplementary Materials**

*S1.1 Tissue Preparation*

To obtain the tissues utilized in this study, whole porcine hearts were harvested and randomly selected from a local abattoir (Hormel Food Corporation, USA). Pericardial tissue was separated from the heart and either decellularized and sterilized following our procedure or glutaraldehyde treated [1], [2]. Additionally, native valves were excised and set aside for mechanical testing. Pericardium was decellularized over 2 days utilizing constant agitation in a cocktail of Sodium dodecyl sulfate (SDS), DNase, and diH2O. This was then washed in a solution of 2% DNase, Tris buffer, MgCl2, 1% peroxyacetic acid (PAA), and phosphate buffered solution (PBS) over 2 days. The decellularized pericardium was sterilized using supercritical carbon dioxide (NovaSterilis, Inc., USA) [1]. For the glutaraldehyde fixed pericardium, the tissues were cleaned and trimmed to size, and the excess fatty tissue removed. Gluteraldehyde was added to PBS at 1.25% and the tissue stored at 4°C for 24 hours. The tissue was then transferred to a new container, with a solution of 0.5% glutaraldehyde in PBS, and stored at room temperature.

*S1.1 Stretch and Stress Measurement*

Cyanoacrylate glue and graphite were combined, and nine small markers for tracking were placed on the center region of the sample by carefully dipping a needle tip into the mixture and manually applying small dots to the tissue taking care to minimize the effect of the glue on the tissue mechanical response. The displacement of the markers was recorded using a Basler Ace camera (Basler, Ahrensburg, Germany) with frame rates up to 510 frames per second and a HP series fixed focal length lens (Basler, Ahrensburg, Germany). The markers were tracked using the TrackMate plug-in within Fiji software [3], [4]. The components of the deformation gradient tensor were then found using finite element shape functions described in Sommer et al. [5]. For brevity these have been excluded here.

For stress measurement, it has been shown previously that due to the limited contralateral forces transferred onto the sample by the rake system shear forces are on the sample are negligible and consequently have little influence on fitted constitutive model parameters [6]. This was confirmed by the low shear strains measured during testing and as such we opted to assume shear forces were insignificant. Furthermore by assuming zero stress in the x_3_ direction the first Piola-Kirchoff (PK1) stress tensor was simplified to

|  | $\mathbf{P}\boldsymbol{=}\left[ \begin{matrix} \frac{f_{1}}{L_{2}T} & 0 & 0 \\ 0 & \frac{f_{2}}{L_{1}T} & 0 \\ 0 & 0 & 0 \end{matrix} \right].$ | (1) |
| --- | --- | --- |

Where $T$ is the sample thickness, $f_{1}$and $f_{2}$ are the measured forces and, $L_{1}$ and $L_{2}$ are the sample length along x_1_ and x_2_ respectively. $T$was recorded prior to testing using a digital thickness gauge (Mitutoyo, Japan) and $L_{1}$ and $L_{2}$ recorded by measuring the distance between the rake pairs. This measurement was performed in ImageJ by measuring the distance between the rakes in the first frame of the test video (after the preload) and converting the measured distance in pixels to mm using a reference length. Additionally, the deformation gradient was also then simplified to

| $\mathbf{F}\boldsymbol{=}\left[ \begin{matrix} \lambda_{1} & 0 & 0 \\ 0 & \lambda_{2} & 0 \\ 0 & 0 & \lambda_{1}^{-1}\lambda_{2}^{-1} \end{matrix} \right],$ | (2) |
| --- | --- |

where $\lambda_{1}$ and $\lambda_{2}$ are the principal stretches in the first and second principal directions.

*S1.2 Statistical Analysis*

To compare the mechanical data between the four strain-rates (in each testing direction) the normalized stress was compared for a given stretch value. Previous studies have compared curve gradient at predetermined stresses to evaluate differences in rate dependence but this was not utilized in this study due to the closeness of the curves which limited the comparison with that method [7]. For each sample the stress was acquired at $\lambda_{i}^{C}$ and $\lambda_{i}^{C}/2$ for all strain rates, where $\lambda_{i}^{C}$ is the lowest maximum stretch for that sample in each principal direction defined for equibiaxial testing as

| $\lambda_{i}^{C}=\min[\max\left( \boldsymbol{\lambda}_{i}^{0.001} \right),\max\boldsymbol{(\lambda}_{i}^{0.01}),\max\boldsymbol{(\lambda}_{i}^{0.1}),\max\boldsymbol{(\lambda}_{i}^{1})] i=1,2.$ | (3) |
| --- | --- |

Where $\boldsymbol{\lambda}_{i}^{0.001},\boldsymbol{\lambda}_{i}^{0.01},\boldsymbol{\lambda}_{i}^{0.1}$ and $\boldsymbol{\lambda}_{i}^{1}$ are vectors of the stretches at each strain rate in each principal direction $i$. For testing at the 3:1 ratio $\lambda_{1}^{C}=\min[\max\left( \boldsymbol{\lambda}_{1}^{0.001} \right),\max\boldsymbol{(\lambda}_{1}^{0.01}),\max\boldsymbol{(\lambda}_{1}^{0.1})]$ and $\lambda_{2}^{C}=\min[\max\left( \boldsymbol{\lambda}_{1}^{0.003} \right),\max\boldsymbol{(\lambda}_{1}^{0.03}),\max\boldsymbol{(\lambda}_{1}^{0.3})]$. The stress was then normalized by the maximum stress for the 0.001 s^-1^ data-set to account for variations in stress magnitude between samples. Following a Kolmogorov-Smirnov test, all data-sets returned a normal distribution thus a one-way ANOVA followed by a multi-comparison test was used to compare the data. Finally, to determine if the relaxation was anisotropic a Student’s unpaired t-test was used to compare the stress at 300 s between each test direction.

S1.3 *Constitutive Modeling*

The static mechanical response was represented by fitting to an anisotropic hyperelastic constitutive model. For this we chose the Gasser-Ogden-Holzapfel model which takes the form of

|  | $W\left( \mathbf{C}\boldsymbol{,}\mathbf{H}_{i} \right)=W_{m}+\sum_{i=1,2} W_{fi} ,$ | ( 4 ) |
| --- | --- | --- |

where $W_{m}$ is the isotropic strain energy for the ground matrix while $W_{fi}$is the anisotropic strain energy of the two families of collagen fibers [8]. These respective components are given as

|  | $W_{m}\left( \mathbf{C} \right)=\frac{\mu}{2}\left( \lambda_{1}^{2}+\lambda_{2}^{2}+\lambda_{3}^{2} -3 \right),$ | ( 5 ) |
| --- | --- | --- |

and

|  | $W_{fi}\left( \mathbf{C}\boldsymbol{,}\mathbf{H}_{i} \right)=\frac{k_{1}}{{2k}_{2}}\text{[}\exp{(k}_{2}E_{i}^{2})-1\text{]},$ | ( 6 ) |
| --- | --- | --- |

where

|  | $E_{i}=\mathbf{H}_{i}:\mathbf{C}-1, \mathbf{H}_{i}\boldsymbol{=}\kappa\mathbf{I}+\left( 1-3\kappa\right)\left( \boldsymbol{a}_{i}\otimes\boldsymbol{a}_{i} \right).$ | ( 7 ) |
| --- | --- | --- |

Here $\lambda_{1},\lambda_{2}$ and $\lambda_{3}$ are the principal stretches with the mean collagen fiber direction $\theta$ in the reference configuration characterized by unit vectors $\boldsymbol{a}_{i}$. The fiber directions are defined by $\boldsymbol{a}_{1}=\left[ \cos\left( \theta\right)\sin\left( \theta\right) 0 \right]$ and $\boldsymbol{a}_{2}=\left[ -cos \left( \theta\right)\sin\left( \theta\right) 0 \right]$ in the x_1_ direction and $\boldsymbol{a}_{1}=\left[ \sin\left( \theta\right) \cos\left( \theta\right) 0 \right]$ and $\boldsymbol{a}_{2}=\left[ -sin \left( \theta\right) \cos\left( \theta\right) 0 \right]$ in the x_2_ direction. The additional parameters are $\mu$, the shear modulus, $k_{1}$, a stress-like parameter and $k_{2}$, a dimensionless parameter. The second order tensors $\mathbf{C}$ and $\mathbf{I}$ are the right Cauchy-Green deformation tensor and second-order identity tensor respectively. The statistical parameter $\kappa\in\left[ 0,\frac{1}{3} \right]$ is the degree of fiber dispersion such that complete alignment of fibers was described by $\kappa=0$ and full dispersion, resulting in isotropy, by $\kappa=\frac{1}{3}$.

The error between experimental and analytical results was minimized using the MATLAB optimization toolbox (Matlab v.2016a, MathWorks, Natick, Ma) for a planar biaxial condition of standard parameter fitting. This can be represented as

|  | $\boldsymbol{x=}\underset{\boldsymbol{x}}{\mathrm{argmin}}\text{[} \vec{P}_{11}^{eq}{,\vec{P}}_{22}^{eq}{,\vec{P}}_{11}^{3:1}{,\vec{P}}_{22}^{3:1}\text{]}$ | ( 8 ) |
| --- | --- | --- |

with

|  | $P_{ii}^{eq}(t)=\vert\vert{P_{ii}^{mod}(t)-(t)P_{ii}^{exp}(t)\left\vert\right\vert^{eq}}$ | ( 9 ) |
| --- | --- | --- |

and

| $P_{ii}^{3:1}(t)=\vert\vert{P_{ii}^{mod}(t)-g_{i}(t)P_{ii}^{exp}(t) \vert\vert^{3:1}}.$ | ( 10 ) |
| --- | --- |

Here $P_{ii}^{mod}$ and $P_{ii}^{exp}$ are the diagonal components of the PK1 stress tensor for the analytical model and the mean experimental data respectively and $\boldsymbol{x}$ is a vector of the model parameters. $\vec{P}_{ii}^{eq}$ and $\vec{P}_{ii}^{3:1}$ are vectors of 1^st^ and 2^nd^ PK1 principal stresses. Upper and lower bounds were applied to limit the fitted parameters to be physically plausible and fits were performed to all testing conditions simultaneously to improve the reproducibility and accuracy of the fitted parameters by minimizing the potential solutions that are below the optimization tolerance. Additionally, a multi-start wrapper was used for this curve fitting which reduces the optimum solution dependence on starting parameters seen when fitting hyperelastic materials [9]. The average experimental data was chosen rather than averaging results to reduce the number of fittings to be performed and to improve accuracy as it has been shown that average constitutive parameters are not guaranteed to represent average behavior [10].

*S1.4 Constitutive Modeling Results*

The fitted parameters to the static data are given in supplementary table I with the root-mean-square-error (RMSE). The native tissues have the lowest $\mu$, $k_{1}$, and $k_{2}$ while the decellularized sterilized pericardium has an equally high $\mu$ as the fixed pericardium and higher $k_{1}$ and $k_{2}$. The mean experimental data at the slowest strain rate and the associated fits to this data are shown in supplementary figure 2.

*S1.5 Tissue Load/Unload Curves*

The loading/unloading behavior of the three biological materials can be seen in supplementary figure 3. It can be seen that the loading and unloading curves follow different paths for all tissues and loading speeds. However, the area between the curves is larger for the faster strain rates implying rate dependency in this behavior as noted elsewhere [11]. Like for the relaxation testing this appears to be in contrast to results seen for the rate dependency testing. Therefore, it may be that the same mechanisms identified by Stella et al. to explain the differing viscoelastic behavior between rate dependent and relaxation testing also explain the disparities in viscoelastic responses between the rate-dependent and load/unload data seen here [12].

[1] R. S. Hennessy *et al.*, “Supercritical Carbon Dioxide–Based Sterilization of Decellularized Heart Valves,” *JACC Basic to Transl. Sci.*, vol. 2, no. 1, pp. 71–84, 2017.

[2] R. S. Hennessy *et al.*, “Recellularization of a novel off-the-shelf valve following xenogenic implantation into the right ventricular outflow tract,” *PLoS One*, vol. 12, no. 8, Aug. 2017.

[3] J.-Y. Tinevez *et al.*, “TrackMate: An open and extensible platform for single-particle tracking.,” *Methods*, vol. 115, pp. 80–90, 2017.

[4] J. Schindelin *et al.*, “Fiji: an open-source platform for biological-image analysis,” *Nat. Methods*, vol. 9, no. 7, pp. 676–682, Jul. 2012.

[5] G. Sommer *et al.*, “Quantification of Shear Deformations and Corresponding Stresses in the Biaxially Tested Human Myocardium,” *Ann. Biomed. Eng.*, vol. 43, no. 10, pp. 2334–2348, Oct. 2015.

[6] H. Fehervary, M. Smoljkić, J. Vander Sloten, and N. Famaey, “Planar biaxial testing of soft biological tissue using rakes: A critical analysis of protocol and fitting process,” *J. Mech. Behav. Biomed. Mater.*, vol. 61, pp. 135–151, 2016.

[7] A. Anssari-Benam, Y. Tseng, G. A. Holzapfel, and A. Bucchi, “Rate-dependency of the mechanical behaviour of semilunar heart valves under biaxial deformation,” *Acta Biomater.*, vol. 88, pp. 120–130, Apr. 2019.

[8] T. C. Gasser, R. W. Ogden, and G. A. Holzapfel, “Hyperelastic modelling of arterial layers with distributed collagen fibre orientations.,” *J. R. Soc. Interface*, vol. 3, no. 6, pp. 15–35, Feb. 2006.

[9] R. W. Ogden, G. Saccomandi, and I. Sgura, “Fitting hyperelastic models to experimental data,” *Comput. Mech.*, vol. 34, no. 6, pp. 484–502, 2004.

[10] D. Robertson and D. Cook, “Unrealistic statistics: How average constitutive coefficients can produce non-physical results,” *J. Mech. Behav. Biomed. Mater.*, vol. 40, pp. 234–239, 2014.

[11] Z. Wang, M. J. Golob, and N. C. Chesler, “Viscoelastic Properties of Cardiovascular Tissues,” in *Viscoelastic and Viscoplastic Materials*, InTech, 2016.

[12] J. A. Stella, J. Liao, and M. S. Sacks, “Time-dependent biaxial mechanical behavior of the aortic heart valve leaflet.,” *J. Biomech.*, vol. 40, no. 14, pp. 3169–77, 2007.

**Supplementary Figure 1**. First Piola-Kirchhoff (PK1) stress versus stretch curves for mean native aortic valve cusp data for the first loading protocol (10% applied equibiaxial strain; N-1) and the second loading protocol (30% applied strain in x_1_ and 10% applied strain in x_2_; N-2). Mean glutaraldehyde fixed (G-1 and G-2) and decellularized-sterilized pericardium (DS-1 and DS-2) curves are also shown. Purple diamonds illustrate points where stress was found at $\lambda_{c}$. Error bars are displayed at 5 increments.

**Supplementary Figure 2.** First Piola-Kirchhoff (PK1) stress versus stretch curves for mean data at the slowest strain rate and the fitted Gasser-Ogden-Holzapfel model curves for all heart valve biomaterials evaluated.

**Supplementary Figure 3.**  Loading and unloading curves detailing first Piola-Kirchhoff (PK1) stress versus stretch for mean native aortic valve cusp data for the first loading protocol (10% applied equibiaxial strain; N-1) and the second loading protocol (30% applied strain in x_1_ and 10% applied strain in x_2_; N-2). Mean glutaraldehyde fixed (G-1 and G-2) and decellularized-sterilized pericardium (DS-1 and DS-2) curves are also shown. Purple diamonds illustrate points where stress was found at $\lambda_{c}$.
